# Hemoglobin decline in cancer patients receiving chemotherapy without an erythropoiesis-stimulating agent

**DOI:** 10.1007/s00520-012-1617-2

**Published:** 2012-10-25

**Authors:** Robert Pirker, Melissa Pirolli, Jane Quigley, Scott Hulnick, Jason Legg, Helen Collins, Johan Vansteenkiste

**Affiliations:** 1 Medical University Vienna, Währinger Gürtel 18-20, 1090 Vienna, Austria; 2 SDI Health, One IMS Drive, Plymouth Meeting, PA 19462 USA; 3 Amgen Inc., One Amgen Center Drive, Thousand Oaks, CA 91320 USA; 4 University Hospital Gasthuisberg, Herestraat 49, 3000 Leuven, Belgium; 5 Department of Medicine I, Medical University Vienna, Vienna, Austria

**Keywords:** Anemia, Hemoglobin, Chemotherapy, Transfusion, Elderly

## Abstract

**Purpose:**

The aim of this study was to examine the rate and timing of hemoglobin decline from <10 g/dL to <9 g/dL in cancer patients receiving chemotherapy.

**Methods:**

Pooled data from the placebo arms of six randomized, controlled trials (RCTs) of darbepoetin alfa and data from an aggregated US community oncology clinic electronic medical records (EMR) database were analyzed. Patients had baseline hemoglobin ≥10 g/dL (RCTs) or baseline hemoglobin between ≥10 g/dL and <11 g/dL (EMR episodes) that declined to <10 g/dL at least once during the study period. The proportion of patients/episodes with hemoglobin decline to <9 g/dL by 3, 6, and 9 weeks without erythropoiesis-stimulating agents was estimated from data in each of the data sources, as was the rate of transfusions in the RCTs.

**Results:**

Data from 411 patients receiving placebo in the RCTs and 10,523 patients (10,942 episodes) in the EMR database were analyzed. Forty percent and 35 % of RCT patients and EMR episodes, respectively, had a hemoglobin decline from <10 g/dL to <9 g/dL at week 3, 54 % and 43 % at week 6, and 58 % and 46 % at week 9. Of patients in the RCTs, 43 % required an RBC transfusion.

**Conclusions:**

Hemoglobin can rapidly decline in cancer patients receiving chemotherapy with hemoglobin levels around 10 g/dL, particularly in patients ≥65 years of age. The rapid rate of hemoglobin decline in these patients should be considered for optimal anemia management.

## Introduction

Anemia is common in cancer patients receiving chemotherapy and is associated with poor clinical outcomes [1]. Chemotherapy-induced anemia can be treated with erythropoiesis-stimulating agents (ESAs), red blood cell (RBC) transfusions, or both. According to current ESA labels, treatment with ESAs in patients receiving chemotherapy should not be considered until hemoglobin levels are less than 10 g/dL in the USA or at or below 10 g/dL in the EU [2–4]. However, ESAs take time to induce a hemoglobin response and therefore are not suitable for patients who require immediate correction of anemia [5]. Studies have suggested that initiating an ESA when hemoglobin is between 9 g/dL and 10 g/dL results in fewer transfusions compared with initiating an ESA when hemoglobin is <9 g/dL [6–8]. However, not all patients whose hemoglobin is in the 9 g/dL to 10 g/dL range will continue to fall to levels of <9 g/dL, and because ESAs have risks, the decision of when to initiate an ESA is partially informed by the rate of hemoglobin decline and the likelihood that the patient will require a transfusion if they do not receive the ESA. Few data are available regarding the proportion of patients with hemoglobin in the range of 9 g/dL to <10 g/dL who will experience a hemoglobin decline to <9 g/dL or the rate at which the decline occurs. A better understanding of factors that influence the rate of hemoglobin decline from 10 g/dL to 9 g/dL may assist oncologists in optimizing the use of ESAs in patients receiving chemotherapy.

We examined rates of hemoglobin decline using two types of data: (1) from patients enrolled in randomized, placebo-controlled clinical trials (RCTs) and (2) from community oncology clinic electronic medical records (EMRs). RCTs with placebo control are usually considered the gold standard of clinical evidence; however, results from these trials, which were initiated in the late 1990s, may not reflect real-world contemporary oncology care in 2012. The purpose of this analysis was to estimate the proportion of patients from pooled data from six RCTs whose hemoglobin declined from a level of <10 g/dL to <9 g/dL and the proportion of patients who further declined to a level that required a transfusion in the absence of treatment with an ESA. As these data were up to 10 years old, we wanted to determine if these results would also be applicable to a contemporary USA patient population and therefore performed the same analysis using data from an EMR database; transfusions are not routinely collected in outpatient EMRs, so the transfusion endpoint could not be evaluated in the patients in the EMR database.

## Patients and methods

### Study design

We analyzed data from two sources: pooled data from the placebo arms of six RCTs of darbepoetin alfa and data from an aggregated US community oncology clinic EMR database maintained by SDI Health. The EMR database included 385,000 annual cancer patients from the USA who had documented hemoglobin values. Data used in this analysis were obtained from 63 outpatient oncology practices.

### Patients

The darbepoetin alfa RCTs included patients with solid tumors [9], lung cancer [10, 11], lymphoproliferative malignancies [12, 13], and multiple tumor types [14]. The subset of placebo patients in these studies who had a baseline hemoglobin ≥10 g/dL and reported at least one hemoglobin value <10 g/dL during the study treatment period (length of trial was 16 weeks for five RCTs and 24 weeks for one RCT) were included in the analysis. In the original studies, patients had a baseline hemoglobin level of ≤11.0 g/dL in four studies [9, 10, 12, 13], <11.0 g/dL in one study [14], and ≥9 g/dL in one study [11].

To closely mirror the criteria of the RCTs, eligible patients in this analysis from the EMR database were ≥18 years old with nonmyeloid malignancies and index hemoglobin ≥10 g/dL and <11 g/dL on or after the start of the chemotherapy episode, with an index hemoglobin date between August 1, 2008 and June 26, 2010. EMR-eligible patients also had received a myelosuppressive chemotherapy doublet (two chemotherapy drugs) between August 1, 2008 and June 26, 2010. Patients could be on any cycle of their chemotherapy regimen as long as they received ≥2 additional chemotherapy cycles at ≤35-day intervals after their index hemoglobin level of 10 g/dL to 11 g/dL. Chemotherapy episodes were re-indexed when the hemoglobin level was <10 g/dL to estimate the proportions of episodes and patients that further declined to hemoglobin <9 g/dL by 3, 6, and 9 weeks without ESA therapy.

EMR-eligible patients could not have received an ESA within 9 weeks before the date when hemoglobin was 10 g/dL to 11 g/dL or at any time during the 18-week study period unless hemoglobin was <9 g/dL. The doublet chemotherapy regimen contained one drug from one of the following categories: anthracycline (doxorubicin, epirubicin, mitoxantrone, liposomal doxorubicin), taxane (paclitaxel, docetaxel, nanoparticle albumin-bound [nab]-paclitaxel), platinum (cisplatin, carboplatin, not oxaliplatin), or gemcitabine. These doublets were chosen to be consistent with a prior non-Amgen publication that analyzed chemotherapy-induced anemia prevalence and incidence in the USA [15]. Chemotherapy was administered at ≤35-day intervals during the first 9 weeks of the study period or until hemoglobin declined to <9 g/dL, whichever occurred first.

### Study endpoints

For patients enrolled in the RCTs, study endpoints included the occurrence of hemoglobin decline from <10 g/dL to <9 g/dL or transfusion by weeks 3, 6, and 9 after hemoglobin <10 g/dL was reached. For patients included in the EMR database, study endpoints were the proportion of patients with hemoglobin decline from the 10 g/dL to 11 g/dL range to <10 g/dL within 3, 6, and 9 weeks and of those episodes with hemoglobin <10 g/dL, further hemoglobin decline to <9 g/dL within 3, 6, and 9 weeks. EMR databases do not reliably capture transfusions that occur outside the clinic setting, and transfusions were therefore not evaluated for patients in the EMR database.

### Statistical considerations

Kaplan–Meier point estimates and associated 95 % confidence intervals (CIs) were provided for time-to-event analyses for patients enrolled in the RCTs. Data were stratified by sex, age (<65 and ≥65 years), tumor type, and chemotherapy type (platinum and other). EMR data were also stratified by sex, age, tumor type, and chemotherapy type (platinum, taxane, anthracycline, or gemcitabine). Index hemoglobin date was defined as the date of first qualifying hemoglobin level (≥10 g/dL and <11 g/dL within 7 days of chemotherapy administration). Index chemotherapy date was defined as the date chemotherapy was received.

## Results

### Patients

This analysis included 411 placebo patients who had baseline hemoglobin ≥10 g/dL and at least one hemoglobin value <10 g/dL enrolled in RCTs and 10,523 patients (representing 10,942 chemotherapy episodes) with hemoglobin ≥10 g/dL and <11 g/dL in the EMR database. Approximately half of the patients enrolled in the RCTs were men (59 %) and patients represented in the EMR database were predominantly women (72 %) (Table 1). Similar proportions of patients were ≥65 years of age (43 % of patients in the RCTs and 39 % of patients in the EMR database). Tumor types differed between the RCT and EMR patients; the predominant tumor types were lung cancer and breast cancer in the RCTs and EMR database, respectively. Chemotherapy regimens differed between the two sets of patients; most (78 %) patients in the RCTs received platinum-based regimens compared with 58.6 % of patients in the EMR database. Additionally, 66.4 % of patients in the EMR database received taxane-based chemotherapy (patients in the EMR database could be counted in more than one chemotherapy category; e.g., carboplatin and paclitaxel would be included in both “platinum” and “taxane” categories).

**Table 1:** Patient demographics and disease characteristics at baseline

	RCT patients^a^	EMR patients	
	Placebo (*N* = 411)	All patients (*N* = 10,523)	All episodes (*N* = 10,942)
Women, *n* (%)	168 (41)	7,565 (72)	7,825 (72)
Age, mean years (SD)	62.5 (10.1)		
Age ≥65 years, *n* (%)	175 (43)	4,147 (39)	4,321 (39)
Race, White *n* (%)	397 (97)		
Tumor type, *n* (%)			
Breast	18 (4)	4,353 (41)	4,418 (40)
Gastrointestinal	14 (3)		
Genitourinary	5 (1)		
Gynecologic	12 (3)	899 (9)	961 (9)
Hematologic	56 (14)		
Lung	297 (72)	3,071 (29)	3,257 (30)
Other	9 (2)	2,200 (21)	2,306 (21)
Stage, *n* (%)			
I/II or lower/limited	39 (10)		
III/IV or higher/extensive	361 (88)		
Other	6 (1)		
Unknown	5 (1)		
Chemotherapy regimen, *n* (%)			
Anthracycline		3,017 (29)	3,050 (28)
Taxane		7,075 (67)	7,269 (66)
Platinum		6,100 (58)	6,408 (59)
Gemcitabine		1,465 (14)	1,504 (14)
Hemoglobin, mean g/dL (SD)	11.26 (0.98)		

^a^Includes only patients who had hemoglobin ≥10 g/dL and had hemoglobin <10 g/dL at least once during the study

*SD* standard deviation *RCT* randomized controlled trials *EMR* electronic medical records

### Hemoglobin decline

**Fig. 1:**
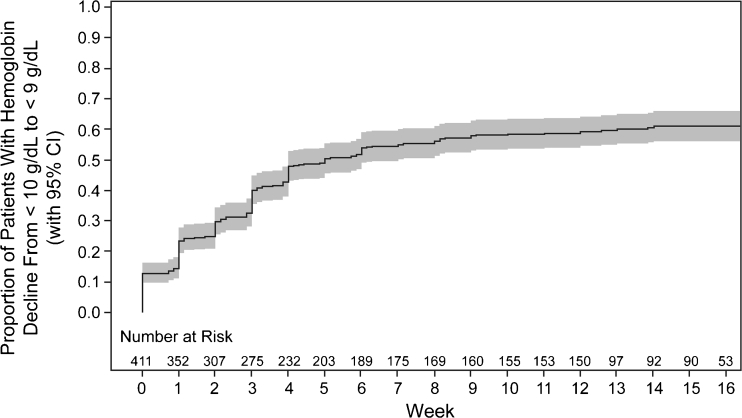
Time to hemoglobin decline from <10 g/dL to <9 g/dL in patients enrolled in RCTs. The Kaplan–Meier analysis of time to hemoglobin decline is shown. *Gray shading* represents 95 % confidence intervals

Similar proportions of patients in the RCTs and EMR database experienced a decline in hemoglobin to <9 g/dL at weeks 3, 6, and 9 (Table 2). Because the majority of placebo patients in the RCTs had received a platinum and/or had lung cancer, patients who received a platinum and/or had lung cancer in the EMR database were analyzed separately; similar proportions of such patients in the RCTs and episodes in the EMR database had a hemoglobin decline from <10 g/dL to <9 g/dL. Most events of hemoglobin decline to <9 g/dL occurred within 3 weeks of chemotherapy, including 40 % of patients in the RCTs and 35 % of episodes in the EMR database. Within 9 weeks, hemoglobin had declined to <9 g/dL in 58 % of patients in the RCTs (Fig. 1) and 46 % of episodes in the EMR database.

**Table 2:** Proportion of patients with hemoglobin decline from <10 g/dL to <9 g/dL

Patients or episodes with hemoglobin decline, % (95 % CI)	RCT patients^a^	EMR episodes^b^		
	Placebo (*n* = 411)	All episodes (*n* = 5,535)	Lung cancer episodes (*n* = 1,804)	Episodes with a platinum doublet (*n* = 3,489)
Week 3	40 % (35, 45)	35 % (34, 37)	42 % (40, 44)	40 % (38, 41)
Week 6	54 % (49, 59)	43 % (41, 44)	48 % (46, 51)	47 % (46, 49)
Week 9	58 % (53, 63)	46 % (44, 47)	51 % (49, 54)	51 % (49, 53)

^a^Values represent Kaplan–Meier percentage estimates

^b^Values represent crude percentages

*RCT* randomized controlled trials, *EMR* electronic medical records *CI* confidence interval

**Fig. 2:**
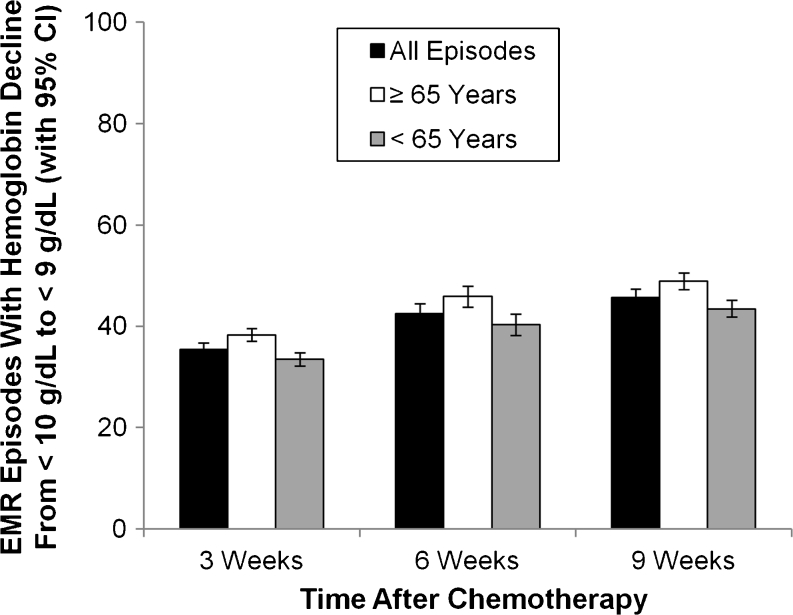
Hemoglobin decline by age group in patients in the EMR database. The proportion of all chemotherapy episodes (*black bars*), episodes for patients ≥65 years of age (*white bars*), and episodes for patients <65 years of age (*gray bars*) with hemoglobin decline from <10 g/dL to <9 g/dL are shown

The percentage (95 % CI) of chemotherapy episodes in the EMR database with a decline in hemoglobin to <9 g/dL within 3 weeks was 38.3 % (6.3, 40.3) for patients 65 years of age and older and 33.5 % (31.9, 35.2) for patients under 65 years of age (Fig. 2). At 9 weeks, the percentages (95 % CI) were 49.0 % (46.9, 51.1) and 43.4 % (41.8, 45.1) for episodes with a decline in hemoglobin to <9 g/dL for patients ≥65 years and <65 years, respectively.

### RBC transfusion

Of the 411 placebo patients enrolled from the RCTs, 178 (43 %) required an RBC transfusion for their anemia (Table 3). By week 9, over one third of patients (39 %) had undergone at least one RBC transfusion.

**Table 3:** Time to first transfusion from first hemoglobin <10 g/dL

Patients transfused^a^, % (95 % CI)	RCT patients (*n* = 411)
Week 3	20 (16, 24)
Week 6	32 (27, 36)
Week 9	39 (35, 44)
Week 12	43 (38, 48)

*RCT* randomized controlled trials, *CI* confidence interval

^a^Values represent Kaplan–Meier percentage estimates

## Discussion

Not all patients on chemotherapy whose hemoglobin has declined to the 9 g/dL to 10 g/dL range will continue to fall to levels of <9 g/dL or require a transfusion. Determining potential risk factors for a hemoglobin decline and the rate of hemoglobin decline would ideally be addressed by a prospective RCT. All the data presented here are retrospective, representing either an unplanned retrospective subgroup analysis of RCT data or retrospective EMR data. As such, the data presented here are hypothesis-generating only.

The data reported here from placebo patients in RCTs suggested that for patients receiving chemotherapy with a hemoglobin decline to <10 g/dL, hemoglobin levels subsequently transition quickly to <9 g/dL. The proportion of chemotherapy episodes where hemoglobin declined to <9 g/dL was similar regardless of age. Consistent with the known enhanced hematotoxicity in elderly patients [16, 17], the hemoglobin decline was slightly more pronounced in patients over 65 years. Despite expected differences between patients enrolled in global RCT trials 10 years ago and patients currently being treated in US community oncology practices, patients with the same tumor types and receiving similar chemotherapy doublets experienced similar rates of hemoglobin decline.

Most of the placebo patients enrolled in the RCTs had lung cancer and/or had received platinum chemotherapy, whereas many cancer types were represented in the EMR database and the most common chemotherapy was taxane. The predominant cancer type represented in the EMR database was breast cancer. Also, EMR patients were treated in 2008–2010 and there may have been some shift in chemotherapy regimens compared with the time frame of the RCTs (late 1990s to early 2000s). Despite differences in study populations, including tumor types and chemotherapy regimens, the proportion of patients who experienced a hemoglobin decline from >10 g/dL to <9 g/dL was quite similar in the RCT and EMR database patients. The decline to <9 g/dL occurred within 3 weeks in 35 % to 40 % of patients and 43 % to 54 % by 6 weeks. In the RCT dataset, the Kaplan–Meier estimate of transfusion rate was 20 % (95 % CI = 16 %, 24 %) within 3 weeks and 32 % (95 % CI = 27 %, 36 %) within 6 weeks.

A strength of this analysis is the consistent results obtained with both the pooled RCT data and the real-world observational EMR data. Limitations of the use of EMR data for our analysis include a potential selection bias due to the voluntary provision of data by oncology practices (treatment patterns may be different in practices that do not participate), and the lack of data for treatments and procedures that are not reimbursable. For example, information on transfusions, inpatient chemotherapy, and oral chemotherapy (for which patients are sent elsewhere) as well as any procedures that occur in a hospital setting (e.g., transfusions) are not included in the EMR database. Therefore, the lack of transfusion data in the EMR did not allow us to determine if the correlation between the RCT and EMR data for hemoglobin decline from <10 g/dL to <9 g/dL also held for the time to first transfusion. Furthermore, this study did not address if specific populations of patients may be more likely to avoid a continued decline in their hemoglobin with a specific intervention, such as an ESA. Patients were required to be iron replete upon entry into all but one of the RCTs; however, although patients with other diagnosis codes for anemia were not included in the EMR data set, as it is an observational database, iron stores were not known.

It is interesting to note the similarity between the results of the RCT and the EMR data base, despite the difference in characteristics of the patients included in the two data sources. Patients enrolled in RCTs represent a select population of cancer patients. These patients tend to have no or few comorbidities and are also more likely to receive their chemotherapy at the full dose and on schedule, although this was not a specific requirement of these ESA clinical trials. In contrast, patients in an EMR database may be more representative of the general population and their treatment patterns may be more representative of community oncology clinic dosages and schedules. The similarity of results observed in the RCT patients and EMR database suggest that the rate of hemoglobin decline in this analysis can be generalized to most cancer patients, regardless of tumor type when receiving common myelosuppressive doublet chemotherapy regimens.

There are currently three options for treating chemotherapy-induced anemia in patients with advanced stage cancer who have hemoglobin levels between 9 g/dL and 10 g/dL. The first option is to wait and watch for continued hemoglobin decline and worsening of anemia symptoms. The second option, for appropriate patients, is to initiate ESA therapy. The third option is to undergo RBC transfusion. Each of these options is associated with benefits and risks. The results of this analysis suggest that for patients with hemoglobin between 9 g/dL and 10 g/dL who are planned to receive further myelosuppressive chemotherapy, the first option of waiting and watching may result in over a third of patients falling to hemoglobin <9 g/dL within 3 weeks. The RCT data further suggest that 32 % will need a transfusion within 6 weeks. Knowing that a potential hemoglobin response to ESAs takes time, waiting and watching may not be the optimal choice for patients with hemoglobin between 9 g/dL and 10 g/dL who want to minimize their risk of transfusion.

In summary, our results suggest that hemoglobin could rapidly decline when hemoglobin levels in cancer patients receiving chemotherapy drop to around 10 g/dL. This decline in hemoglobin was associated with a high rate of RBC transfusions.
